# Molecular Mechanism of Binding between 17β-Estradiol and DNA

**DOI:** 10.1016/j.csbj.2016.12.001

**Published:** 2016-12-12

**Authors:** Tamsyn A. Hilder, Justin M. Hodgkiss

**Affiliations:** a School of Chemical and Physical Sciences, Victoria University of Wellington, Wellington 6040, New Zealand; b Computational Biophysics Group, Research School of Biology, Canberra, ACT 0200, Australia; c The MacDiarmid Institute of Advanced Materials and Nanotechnology, New Zealand

**Keywords:** 17β-estradiol, Estrogen response element, Molecular dynamics, Intercalation

## Abstract

Although 17β-estradiol (E2) is a natural molecule involved in the endocrine system, its widespread use in various applications has resulted in its accumulation in the environment and its classification as an endocrine-disrupting molecule. These molecules can interfere with the hormonal system, and have been linked to various adverse effects such as the proliferation of breast cancer. It has been proposed that E2 could contribute to breast cancer by the induction of DNA damage. Mass spectrometry has demonstrated that E2 can bind to DNA but the mechanism by which E2 interacts with DNA has yet to be elucidated. Using all-atom molecular dynamics simulations, we demonstrate that E2 intercalates (inserts between two successive DNA base pairs) in DNA at the location specific to estrogen receptor binding, known as the estrogen response element (ERE), and to other random sequences of DNA. Our results suggest that excess E2 has the potential to disrupt processes in the body which rely on binding to DNA, such as the binding of the estrogen receptor to the ERE and the activity of enzymes that bind DNA, and could lead to DNA damage.

## 1 Introduction

17β-estradiol (or E2) is a natural steroidal hormone and is also commonly used in therapeutics such as postmenopausal estrogen replacement therapy and the treatment of Alzheimer's disease. Unfortunately, it has also become one of the most widely encountered endocrine-disrupting molecules in the environment [1]. Endocrine-disrupting molecules, either natural or synthetic, can interfere with the hormonal system. For example, they have the potential to cause cancerous tumours, birth defects, and developmental disorders in humans, while low concentrations (ng/L) of natural and synthetic estrogen hormones have been shown to have a harmful effect on fish [2]. Unfortunately, their widespread use in applications such as therapeutics and plastics has resulted in the accumulation of endocrine disrupting molecules in groundwater, rivers and lakes. Environmental exposure to endocrine-disrupting molecules such as E2 has the potential to cause adverse effects to the ecosystem and to humans [2], [3], [4], [5], particularly through their presence in drinking water.

In the presence of normal levels of E2, the consensus response mechanism involves E2 first binding to an estrogen receptor (ER) protein. Then, this E2-ER complex forms a dimer and binds to the estrogen response element (ERE) on the DNA strand to initiate a hormone response. This ERE is a palindromic DNA sequence, and similar sequences have been identified in numerous sequences involving estrogen action such as oxytocin [6]. The E2-ER complex measures both the spacing and helical repeat of it's ERE, thus greatly increasing the specificity of the interaction [7]. A disruption to this spacing and the helical repeat may be sufficient to disturb this very delicate conformational equilibrium and cause unwanted side effects.

In the presence of elevated levels of E2, it has been suggested that E2 can directly interact with the ERE and interfere with normal signalling. Estrogen signalling drives cell proliferation in 60–70% of breast cancers that express the estrogen receptor [8], and anti-estrogen therapy is prescribed to the majority of these patients to prevent breast cancer recurrence. Estrogen exposure is now widely accepted as a risk factor in breast cancer development, but the mechanisms through which estrogens induce breast carcinogenesis have not been fully elucidated [9]. Typically, research has demonstrated that endocrine disrupting molecules, such as bisphenol-A inhibit the hormone binding pocket on the estrogen receptor [10], [11], [12]. However, this may not be the only mechanism by which endocrine disruptors cause harm. Caldon [8] state that high levels of estrogen are a major risk for breast cancer, and that one mechanism by which estrogen could contribute to breast cancer is via the induction of DNA damage. Using mass spectrometry, Heger et al. [9] found that E2 binds to DNA and leads to destabilization of hydrogen bonds between nitrogenous bases of DNA strands resulting in a decrease of their melting temperature. Their results revealed that E2 forms non-covalent physical complexes with DNA, and they suggest that these interactions could trigger mutations leading to unwanted side effects [9]. DNA damage as a result of exposure to E2 has also been demonstrated in barnacle larvae [13] and rodents [14], for example. In rodents, this DNA damage ultimately led to tumours in estrogen-responsive tissues [14]. Zhang et al. [15] demonstrated experimentally that bisphenol-A intercalates between adjacent base pairs of DNA.

Despite the risks associated with estrogen exposure the exact mechanisms by which estrogen contributes to the initiation and progression of breast cancer remains elusive [8]. However, a major mechanism is potentially the induction of DNA damage as estrogen treatment leads to double stranded DNA breaks and genomic instability [8]. We use all-atom molecular dynamics (MD) simulations to elucidate how E2 binds to DNA, and to obtain critical understanding of the effect of this binding on the DNA structure. We demonstrate that excess E2 could disrupt estrogenic processes in our bodies by binding directly to the ERE.

## 2 Material and Methods

### 2.1 System Setup

**Fig. 1:**
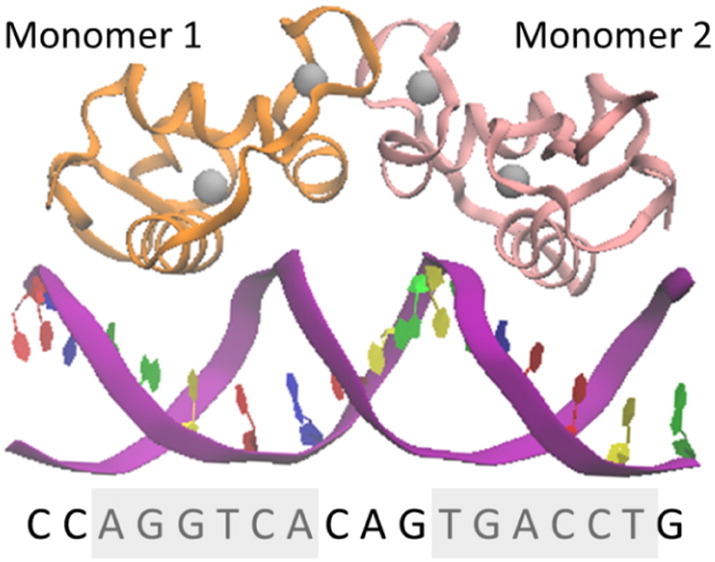
ER dimer-DNA complex. Atomic coordinates are taken from Protein Data Bank (PDB) entry 1HCQ (12) and image is created using VMD (18). Each monomer is represented as a ribbon (orange and pink, respectively). Grey spheres represent the two zinc ions associated with each monomer. The DNA sequence of one strand is given below the ER-DNA complex. The two half-sites are highlighted in grey. Colours are used to help identify DNA backbone (purple), and bases adenosine (blue), guanine (green), thymine (yellow) and cytosine (red). Note that for clarity bases are only shown on the strand of DNA given in the sequence shown. (For interpretation of the references to colour in this figure legend, the reader is referred to the web version of this article.)

The initial coordinates for the ER DNA-binding domain (DBD) were taken from the Protein Data Bank (PDB) with the entry 1HCQ, determined to 2.4 Å resolution [7]. The crystal structure contains the ER symmetric dimer (ER-α, and ER-β) bound to DNA at its palindromic binding site, as shown in Fig. 1. The palindromic binding site consists of two 6 base pair (bp) consensus half sites with 3 intervening bps, illustrated in Table 1. This sequence is referred to as erDNA.

### 2.2 Molecular Docking

**Fig. 2:**
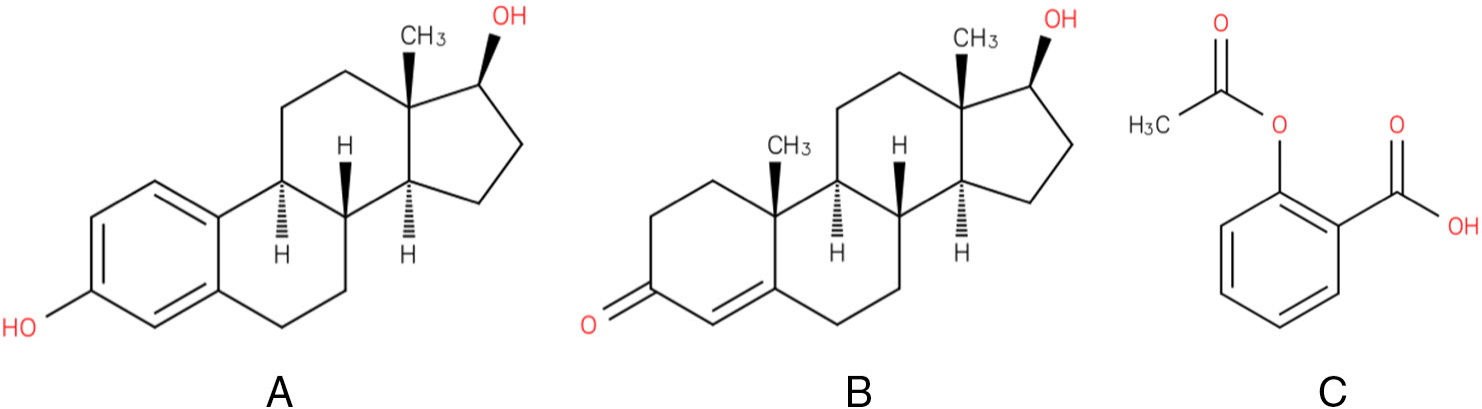
Chemical structure of A) 17β-estradiol (C_18_H_24_O_2_), B) testosterone (C_19_H_28_O_2_), and C) aspirin (C_9_H_8_O_4_). Image obtained from the Chemical Entities of Biological Interest (ChEBI) reference database (17β-estradiol, ChEBI ID: 16,469; testosterone, ChEBI ID: 17,347; aspirin, ChEBI ID: 15,365) (15).

We used the rigid body docking program ZDOCK 3.0.1 [16] to generate a set of conformations of E2 bound to erDNA in the absence of the ER protein. The erDNA sequence in Table 1 was isolated from the ER-DNA complex and the double stranded erDNA was used as input into the rigid docking program ZDOCK [16] to generate a set of likely bound complexes with E2. The atomic coordinates of E2 were obtained from the Protein Data Bank (PDB) entry 1FDS [17], and the chemical structure of E2 is shown in Fig. 2A [18]. Chemicalize.org was used to obtain the atomic partial charges, January 2015, chemicalize.org and ChemAxon (http://www.chemaxon.com). Although ZDOCK is typically used for protein-ligand docking, Fanelli and Ferrari [19] have proven the effectiveness of ZDOCK for DNA-protein docking. We searched the top-100 ranked structures for possible E2-erDNA complexes. The flexibility of the complex is not taken into account in ZDOCK. Therefore, we performed MD simulations to determine the predicted bound state. The highest-ranked docked structure was used as the starting configuration in MD simulations.

### 2.3 Molecular Dynamics Simulations

MD simulations are used to determine the bound configuration of E2-erDNA complex and estimate the strength of binding. All MD simulations are performed using NAMD 2.10 [20] and visualized using VMD 2.9.2 [21]. Throughout, we used the CHARMM36 force field [22], [23], and CHARMM27 force field for nucleic acids [24], [25]. We used TIP3P water, with a time step of 2 fs, at a constant pressure (1 atm), and temperature (310 K). The temperature is below the expected melting temperature of the double stranded erDNA, which is approximately 325.6 K [26]. The E2-erDNA complex was solvated in a water box such that there is a layer of water 20 Å in each direction from the atom with the largest coordinate in that direction. The particle-mesh-Ewald (PME) algorithm was used for the electrostatics with a tolerance of 10^− 6^. The erDNA strand and E2 were initially held fixed to allow the water to equilibrate during the simulation period of 0.15 ns. Unbiased MD simulations were run for 40 ns to determine the stability of the bound complex. In these simulations no constraints are applied. Both the electrostatic and van der Waals non-bonded interaction energies are calculated using the NAMD Energy plugin available in NAMD2.10 [20].

We estimate the free energy of binding for the E2-erDNA complex using free energy perturbation (FEP) method. For FEP, the target is both annihilated and created in both a free and bound state. In other words, the transformation is performed bidirectionally and the forward and backward simulations are combined using the Bennett acceptance-ratio (BAR) estimator of the free energy which corresponds to the maximum likelihood value of the free energy. The ParseFEP plugin is used, with the Gram-charlier order set to 0 to compute the free energy difference between annihilation and creation simulations, and estimate the statistical error [27].

We build two different molecular systems; E2 in a water bath and E2 bound to erDNA, also in a water bath. To avoid any differences in the two simulations the dimensions of the simulation cell are identical for the free and bound state. In both cases a water box of 64 by 64 by 100 Å^3^ is used. The annihilation is performed using 40 λ windows, differing by 0.025. For each window, 5000 fs of equilibration is performed, before ensemble averaging is turned on for a further 20,000 fs. A softcore potential is used to avoid explosively large energy values at each end of the λ scale when E2 is nearly annihilated. This scales down the electrostatic interactions from λ = 0.5 to λ = 1.0, and the van der Waals interactions from λ = 0 to λ = 1.0 for the annihilated molecule. To prevent E2 from moving away it is restrained to stay within its normal fluctuating position from equilibration simulations. The positional restraint results in a loss of translational entropy, Δ*G*_rest_ equal to − 1/β ln (*c*_0_ ∆* v*), where β is equal to 1/*k*_*B*_*T*, Δ*v* is the effective volume sampled by the target and *c*_0_ is the usual standard concentration. The difference between the net free-energy changes for E2 in its free and bound states yields the binding free energy, to which the contribution due to the positional restraint is added, thus

(2) $$∆G_{\mathrm{bind}}=∆G_{\mathrm{free}}-∆G_{\mathrm{bound}}+∆G_{\mathrm{rest}}$$

We can then estimate the dissociation constant of target binding using the relation

(3) $$K_{d}=1/\exp\left(-∆G_{\mathrm{bind}}/k_{B}T\right).$$

The accuracy of MD simulations is dependent on the quality of sampling and the accuracy of the force-field [28]. Two force fields commonly used for simulations of nucleic acid-protein complexes are AMBER [29] and CHARMM [22], [23], [24], [25]. The CHARMM force field has performed well with nucleic acid structural integrity due to its sophisticated atom-based smoothing of electrostatic forces [30]. Galindo-Murillo et al. [31] recently demonstrated the convergence and reproducibility of MD simulations using both AMBER and CHARMM nucleic acid force fields. They suggested that ~ 1 μs length simulation or longer are needed to converge the structural properties of free-DNA in solution, minus the two terminal base pairs at each end [31]. Due to the computational cost we were unable to run our simulations this long. However, investigations into several protein-DNA, ligand-DNA systems have demonstrated the feasibility of the MD simulation approach with smaller time scales [30], [32]. For example, Mukherjee et al. [32] successfully used MD to provide detailed mechanistic insight into the intercalation of the anticancer drug duanomycin into DNA. Moreover, their results using only 7.5 ns MD simulations compare well with experimental results. Laughton and Harris [33] provide a good review relating to the computational simulation of DNA. As a control, we ran an additional simulation of the erDNA strand in the absence of E2 for 40 ns. We then compared the erDNA structures in the presence and absence of E2 to assess the effect of the force field and to determine the effect that E2 has on erDNA structure. As a further assessment of the chosen force field we also ran simulations of the erDNA structure with and without E2 using a recently developed CHARMM force field which was optimized for DNA [34].

To assess the importance of the ERE in the erDNA sequence in binding to E2 we ran two additional simulations. First, we used a DNA random sequence generator to scramble the ERE half site (www.faculty.ucr.edu/~mmaduro/random.htm). This new sequence (rDNA) is illustrated in Table 2. Using 3D-DART we generated a 3D model of this randomised sequence [35]. Second, we examined the binding of testosterone to the original erDNA sequence. The atomic coordinates of testosterone were obtained from the Protein Data Bank (PDB) entry 2Q7I [36], and the chemical structure of testosterone is shown in Fig. 2B [18]. Chemicalize.org was used to obtain the atomic partial charges, January 2015, chemicalize.org and ChemAxon (http://www.chemaxon.com). As described above, we used ZDOCK and MD simulations to examine these additional bound complexes.

Spectroscopic studies have shown that aspirin binds to the minor groove of DNA, but does not intercalate into DNA [37]. Therefore, we also examine the binding of aspirin to the original erDNA sequence to provide a comparison with E2 and testosterone. The atomic coordinates of aspirin were obtained from the Protein Data Bank (PDB) entry 1TGM [38], and the chemical structure of aspirin is shown in Fig. 2C [18]. The atomic partial charges were taken from Jämbeck et al. [39].

Our simulations do not have explicit ions in the system rather a neutralizing background charge is applied [40]. We examine the effect of neutralizing background charge by simulating one of the bound complexes of E2 to erDNA with 34 explicit Na + ions. We find that the effect is negligible and therefore ions are not included in our system.

## 3 Results and Discussion

### 3.1 Molecular Docking

**Fig. 3:**
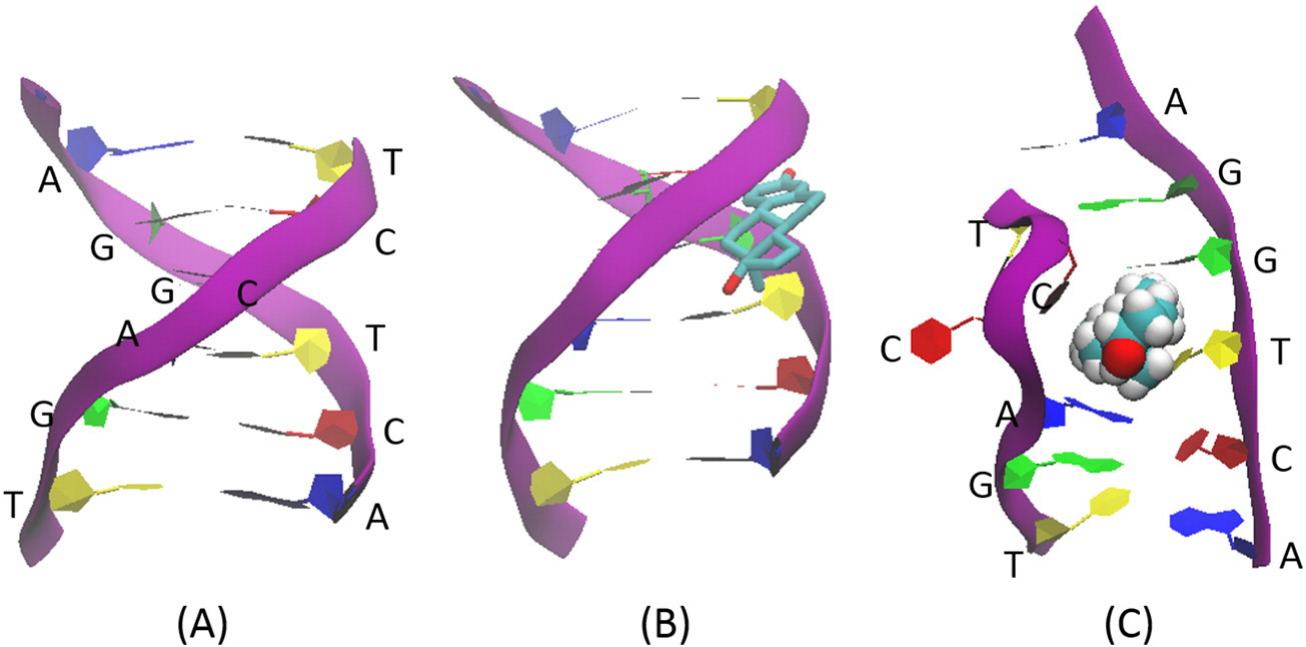
Palindromic consensus half site with E2 (A) absent, (B) bound complex from ZDOCK, and (C) bound complex after 40 ns MD simulations. Note that only a portion of the DNA strand is shown, and is the location of the palindromic consensus half site. Colours are used to help identify backbone (purple), adenosine (blue), guanine (green), thymine (yellow) and cytosine (red). For clarity, in (B) E2 is shown in licorice with hydrogen atoms removed but as VdW spheres in (C). (For interpretation of the references to colour in this figure legend, the reader is referred to the web version of this article.)

For the binding of E2 to erDNA, only two bound complexes are observed in the top-100 ranked structures from our rigid body docking simulations. These two complexes are bound at the location of the two palindromic half sites (Table 1) previously identified as important for binding with ER [7]. Specifically, in 67% of the complexes E2 is bound at G5 and T6, and in the remaining 33% of complexes E2 is bound at A14 and C15 (arrows shown in Table 1). These locations are identical due to DNA symmetry and therefore only one is included in subsequent MD simulations. The bound complex is shown in Fig. 3B.

For the binding of E2 to rDNA, there are two main bound complexes observed in the top-100 ranked structures. Specifically, E2 is bound to C8 and C9 in 51% of the complexes, and G12 and C13 in 48% of the complexes. Both of these complexes are included in subsequent MD simulations.

Two bound complexes are also observed in the top-100 ranked structures for the binding of testosterone to erDNA. As with E2 binding, these two complexes are bound at the location of the two palindromic half sites. Specifically, in 53% of the complexes testosterone is bound at G5, and in the remaining 47% of complexes testosterone is bound at C16. Again, due to symmetry only one of these complexes is included in subsequent MD simulations.

Similarly, two bound complexes are observed in the top-100 ranked structures for the binding of aspirin to erDNA at the location of the two palindromic half sites. Specifically, aspirin is bound at G4 and G5 in 67% of the complexes and in the remaining 33% is bound to C15 and C16. Therefore, due to symmetry we consider only one of these complexes in subsequent MD simulations.

### 3.2 MD Simulations

**Fig. 4:**
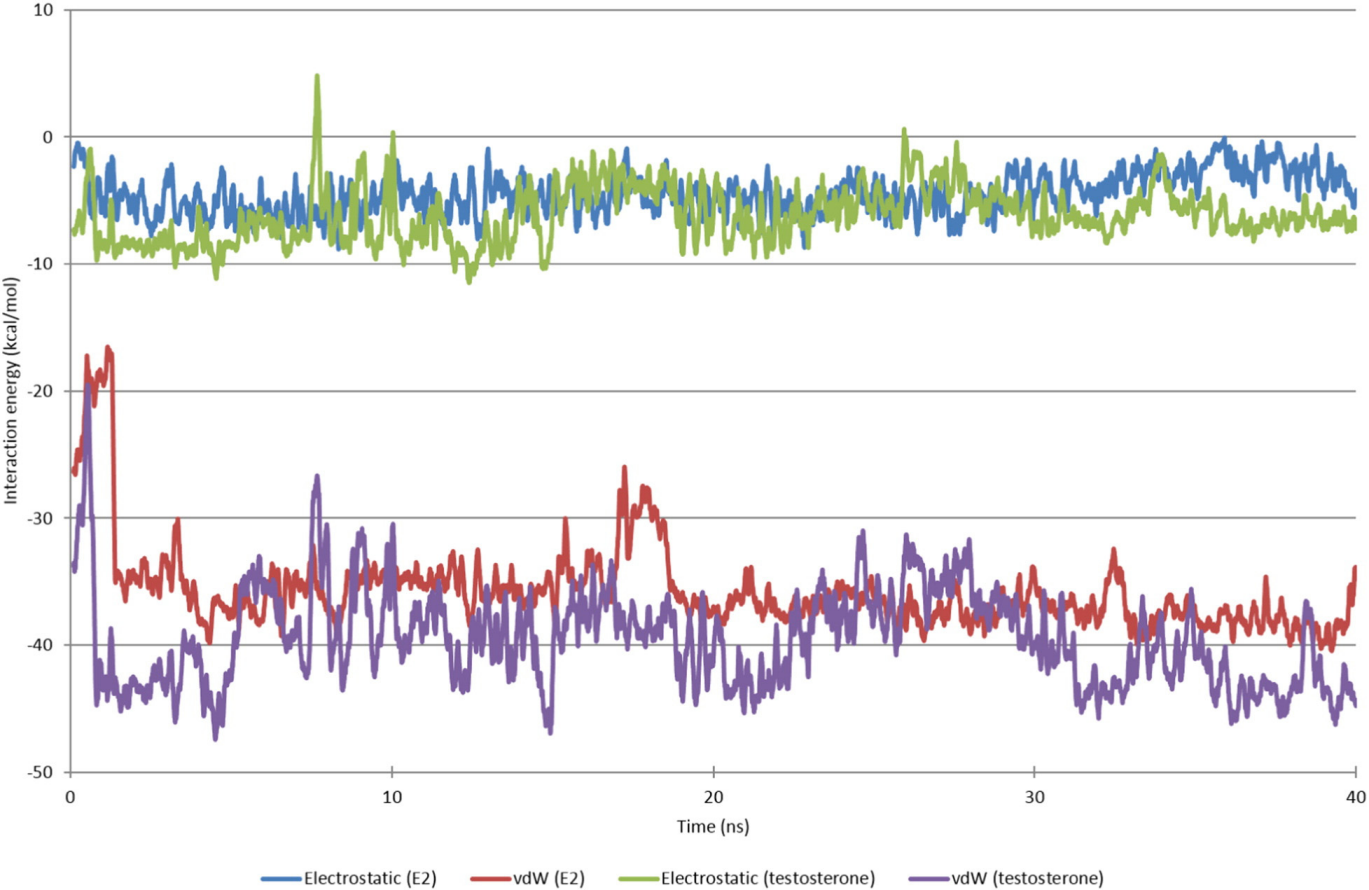
Electrostatic and van der Waals (VdW) interaction energies between 17β-estradiol and testosterone, and the ds-erDNA. Moving average is displayed, averaging over 20 data points.

After 40 ns E2 remains bound to both the erDNA and one of the rDNA sequences. As shown in Fig. 3C, E2 is inserted into the erDNA strand at the centre of the palindromic half site. In agreement with the mass spectrometry results of Heger et al. [9], we find evidence for the plausibility of non-covalent bonding that is dominated by van der Waals interactions. As shown in Fig. 4, electrostatic interactions between E2 and the erDNA strand are approximately − 4.47 ± 2.77 kcal/mol, whereas van der Waals interactions are approximately − 35.69 ± 3.98 kcal/mol. Our results provide detailed molecular insight into this interaction.

**Fig. 5:**
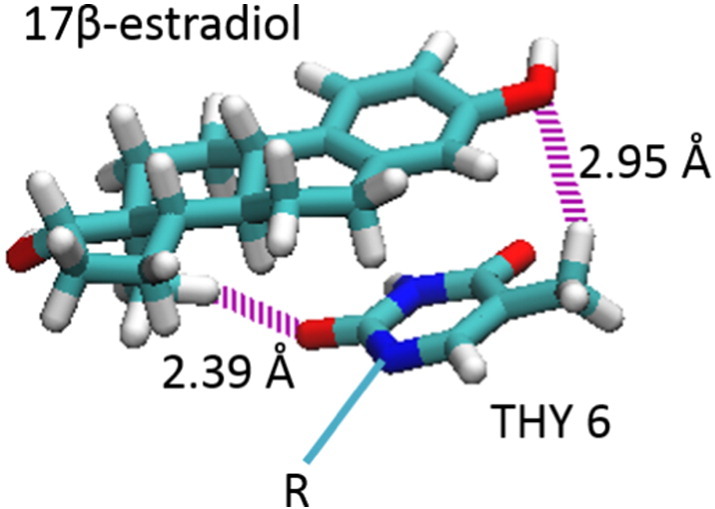
Hydrogen bonding between base T6 and 17β-estradiol.

**Fig. 6:**
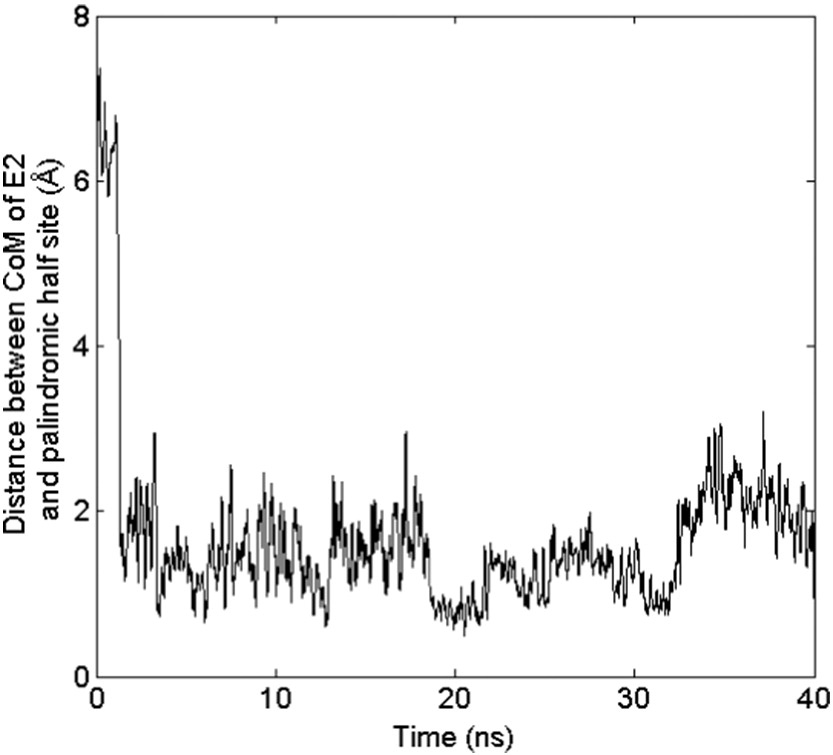
The distance between the centre of mass (CoM) of E2 and the palindromic half site (located at G5/T6) as a function of simulation time. Moving average is displayed, averaging over 20 data points.

For the binding to erDNA, we find that E2 forms aromatic interactions with the thymine base on one strand and the cytosine base on the opposing strand, intercalating between bases G5 and T6. Fig. 5 illustrates the two hydrogen bonds which are formed between the base T6 and E2. A hydrogen bond is assumed to be formed if the donor-acceptor distance is < 3.0 Å and the donor-hydrogen-acceptor angle is ≤ 30°. Both face-to-face and parallel displaced pi stacking is occurring between the bases T6 and C34 with E2, respectively. A pi-pi face-to-face interaction is assumed to be formed if the angle between two aromatic ring planes is less than 30° and the distance between the ring centroids is less than 4.4 Å (https://www.schrodinger.com/kb/1556). The distance between T6 and E2 aromatic ring centroids is 3.98 Å with an angle of 19.4°, so that this interaction can be considered as a face-to-face pi-pi interaction. The interaction of E2 with C34 has a distance between aromatic ring centroids of 4.56 Å and an angle of 76.1°. These values are greater than the requirements for a face-to-face pi-pi interaction, but the offset distance of these aromatic rings is 1.09 Å, so that this interaction can be considered as a parallel displaced pi-pi interaction following the definition given by Hunter and Sanders [41] for an idealized pi atoms and provided in Fig. S1 of the Supporting Information. The Movie S1 in the Supporting Information illustrates E2 moving from its initial ZDOCK bound state to the intercalated state. The binding between G5 and T6 is fairly stable, as shown in Fig. 6 after approximately 2.5 ns there is little fluctuation of the distance between the centre of mass of E2 and the centre of the palindromic half site (1.49 ± 0.69 Å). Furthermore, one cytosine base swings away from the interior of the erDNA strand to accommodate the E2 molecule (see Fig. 3C). The RMSD between the erDNA strand in its bound form and the erDNA crystal structure is 11.68 Å, suggesting that there is considerable deviation from the original crystal structure. Furthermore, the RMSD between erDNA after 40 ns in the presence and absence of E2 is 8.21 Å. The differences in these structures in the vicinity of the half site are illustrated in the Supporting Information Fig. S2.

**Fig. 7:**
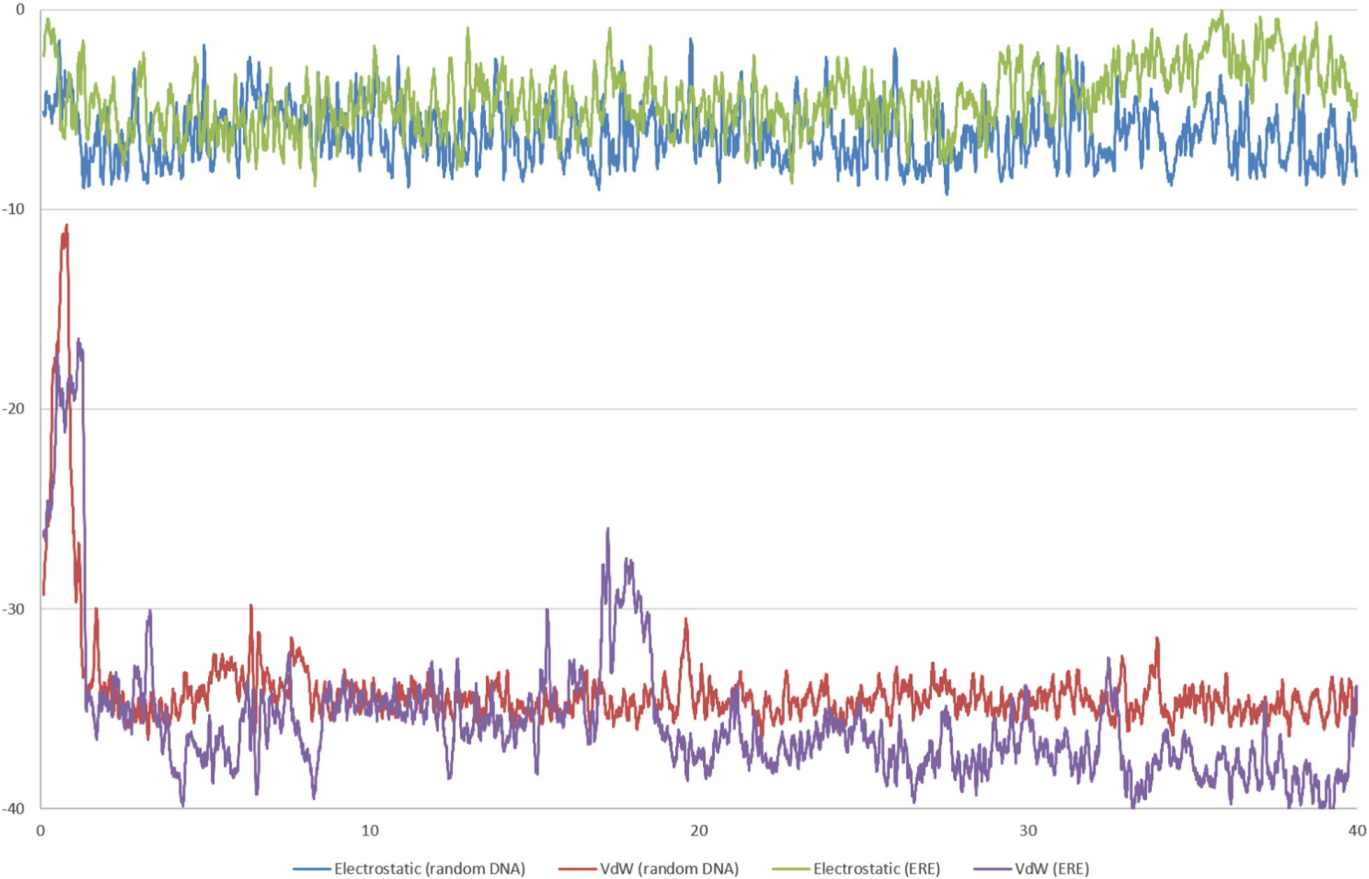
Comparison of the electrostatic and van der Waals (VdW) interaction energies between 17β-estradiol and either the rDNA (random DNA) or erDNA (ERE) strand. Moving average is displayed, averaging over 20 data points.

For the binding to rDNA, we find that E2 forms aromatic interactions with C8 on the first strand and the opposing guanine residues on the opposing strand (G11 and G12 in Table 2). As shown in Fig. 7, the interaction energies are similar magnitude to the binding to erDNA and binding is dominated by van der Waals interactions. The electrostatic interactions between E2 and the rDNA strand are − 6.26 ± 2.64 kcal/mol, whereas the van der Waals interactions are − 34.08 ± 3.19 kcal/mol. This suggests that binding is not specific to the ERE sequence.

We also find that after 40 ns testosterone remains bound to erDNA. Testosterone intercalates between bases G4 and G5. Again, van der Waals interactions dominate with interaction energies a similar magnitude to E2, as shown in Fig. 4. The electrostatic interaction energy between testosterone and erDNA is − 6.15 ± 3.13 kcal/mol, whereas the van der Waals interaction energy is − 33.36 ± 3.42 kcal/mol. This demonstrates that binding is not specific to the hormone E2, and that excess testosterone could have a similar negative impact on DNA.

In our simulations, the hormone (either E2 or testosterone) preferentially binds to the sequence GGT. These bases then stabilize the binding through aromatic interactions either on opposing strands, or in the case of rDNA between the two guanine bases. The anticancer drug daunomycin also intercalates into DNA [32] and has been shown to bind preferentially to the sequence A/T,C,G [42]. Another endocrine disruptor, bisphenol-A has been shown to intercalate between adjacent base pairs of DNA [15]. The presence of aromatic rings in these molecules renders them able to intercalate with the aromatic bases. An aromatic ring is also present in aspirin, but unlike E2 and testosterone we find that aspirin binds to the minor groove of erDNA and does not intercalate into the DNA strand as demonstrated in earlier spectroscopic studies [37]. Furthermore, aspirin does not stay bound to erDNA for longer than 0.5 ns. Prior to unbinding, the average electrostatic and van der Waals interaction energy over the first 0.5 ns is − 11.03 ± 5.84 and − 11.47 ± 2.75 kcal/mol, respectively. Fig. S3 in the Supporting Information illustrates the interaction energy between aspirin and erDNA over the 4 ns simulation.

As shown in Fig. 3 the binding of E2 has opened a gap in the DNA structure and disrupted the DNA structure such that the helical parameters have changed compared to the crystal structure [7]. It is well known that the binding of intercalators to DNA induces conformational changes in DNA structures including the opening of a gap between the flanking bases and an elongation and unwinding of the helical twist [43]. These structural changes affect the biological functions of DNA including the inhibition of transcription, replication, and DNA repair processes, thereby making intercalators potent mutagens and potential antitumor drugs [43]. Destabilization can trigger mutations leading to unwanted side effects [9].

The crystal structure of the erDNA duplex (1HCQ) has a mean helical twist and rise of 35.88° and 3.39 Å, respectively [7]. Using Curves + [44], [45], we obtain local helical parameters of the erDNA structure in the vicinity of binding after 40 ns (i) in the presence of E2, (ii) in the presence of testosterone and (iii) in the absence of any hormone. We also examine the local helical parameters of the erDNA structure after 40 ns in the presence and absence of E2 for the alternative CHARMM force field [34]. In all cases (with and without E2/testosterone) the mean helical twist in the vicinity of binding decreases compared to the crystal structure. Thus, since both our control and bound complexes exhibited unwinding we are unable to comment on whether the binding of these hormones induces unwinding of the DNA duplex.

On the other hand, the mean rise of the equilibrated crystal structure in the absence of any hormone compares well with the unequilibrated crystal structure (3.39 Å) and is 3.5 Å. In contrast, the mean rise in the vicinity of binding in the presence of E2 and testosterone increases to 4.2 Å and 3.9 Å, respectively, demonstrating a lengthening of the DNA duplex compared to the equilibrated crystal structure. Since our results are showing that the mean helical twist decreases regardless of the presence of hormone it is likely that the force field is influencing these structural changes observed in our simulations. We are currently collaborating with quantum chemists to devise a more accurate force field specific for this DNA strand.

### 3.3 Effect on ER Binding

The ER protein is shown to interact with the central four base pairs of the 6 bp half site using four amino acid side chains on the surface of the recognition helix [7]. ER-α and ER-β bind cooperatively to DNA forming a dimer, as shown in Fig. 1. By forming this cooperative dimer, the protein measures both the spacing and helical repeat of its response element, thus greatly increasing the specificity of the interaction [7]. Side chains of the proteins make sequence specific hydrogen bonds to the phosphate backbone of the central 4 base pairs of the 6 bp half site. The dissociation constant of ER-α and ER-β binding to the consensus half site is approximately 0.6 nM and 1.5 nM, respectively [46], [47], equivalent to a binding energy of between − 12.1 and − 12.7 kcal/mol. This binding is specific to the consensus half site as demonstrated by the fact that the dissociation constant of ER-α binding to plasmid DNA is 400-fold higher [47]. Using FEP we obtain a free energy of binding of E2 to erDNA -8.8 ± 1.2 kcal/mol, which corresponds to a dissociation constant of 388 nM.

The binding of E2 (or other hormones such as testosterone) may be sufficient to disturb the position of this very delicate conformational equilibrium. Typically, estrogens exert their biological effects through a direct interaction with the estrogen receptor which then activates the expression of genes encoding proteins with important biological functions. However, research has shown that unbound hormones can readily diffuse into cells. Oren et al. [48] demonstrated that steroid hormones, such as progesterone, testosterone and estradiol freely diffuse across biomembranes and that this diffusion is rapid, ~ 0.01 s to cross a 30 Å thick biomembrane. It is also possible for small molecules, such as hormones, to diffuse into the nucleus, either by passive diffusion [49] or through nuclear pore complexes [50]. Thus, excess E2 could bind to or near the response element prior to ER binding thus disrupting the DNA structure such that the protein spacing does not match the helical repeat of its response element. Alternatively, the portion of DNA structure with E2 bound may be recognized as an ‘error’ which could result in a deletion from the DNA sequence or a frameshift mutation which would again affect the interaction of ER with DNA response element and interfere with signalling and regulation. Both of these possibilities could cause some serious unwanted side effects of E2 accumulation to normal hormone function. Intercalating drugs, such as ethidium bromide, have already been shown to inhibit the interaction of ER with DNA [51]. It is also possible that E2 could intercalate with DNA sequences other than the ERE, since we have demonstrated that E2 also remains bound to the randomised sequence (rDNA), thus damaging other cellular processes such as the activity of enzymes that bind DNA [51]. For example, some anticancer drugs (such as daunomycin) act by intercalating into DNA thus inhibiting the enzyme topoisomerase which stops DNA replication and leads to cell death [32].

## 4 Conclusions

We have demonstrated that E2 can bind to DNA directly and that binding not only occurs at the centre of the ERE half site but can also bind to random DNA sequences. E2 is shown to intercalate between base pairs, forming aromatic interactions with these base pairs. We demonstrate the intercalation results in a lengthening of the DNA duplex, but are unable to comment on the unwinding due to inaccuracies in the force field. In ongoing work we are working with colleagues to develop a more accurate force field to represent specific DNA sequences. We predict that this intercalation will alter the structure of the DNA duplex, and therefore have the potential to affect the biological functions of DNA including the inhibition of transcription, replication, and DNA repair processes [40]. Therefore, excess E2 has the potential to exert some serious side effects such as disruption of ER-DNA binding, DNA damage and possibly the initiation of cancer.

The following are the supplementary data related to this article.

Movie S1 17β-estradiol moving from its initial ZDOCK bound state to its intercalated state from molecular dynamics simulations. 17β-estradiol is shown in licorice with hydrogen atoms removed for clarity. Only the segment of the DNA strand involved in binding is shown. Movie S1

Supplementary material including Figure S1, Figure S2 and Figure S3. Image 1

## Declaration of Interest

The authors declare that there is no conflict of interest.

## Author Contributions

The manuscript was written through contribution of all authors. TAH conducted the simulations.

## Figures and Tables

**Table 1:**
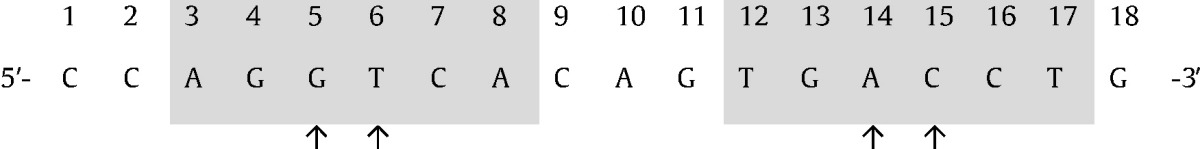
The DNA sequence taken from PDB ID: 1HCQ (12). The two 6 base pair consensus half sites are highlighted in grey. Arrows indicate bases involved in binding to E2.

**Table 2:**
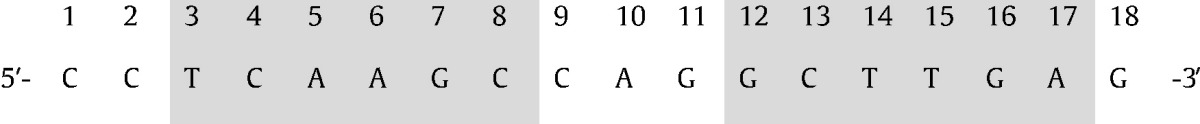
The DNA sequence taken from PDB ID: 1HCQ (12) with the ERE palindromic half-site randomised. The two 6 base pair half sites are highlighted in grey.
