# Supplementary material for: Molecular Mechanism of Binding between 17β-Estradiol and DNA
Source: Comput Struct Biotechnol J. 2016 Dec 12;15:91–7. doi: 10.1016/j.csbj.2016.12.001 (PMC5196241; doi:10.1016/j.csbj.2016.12.001)
Supplement: Supplementary file 2 — Supplementary material including Figure S1, Figure S2 and Figure S3. [file mmc2.docx]

**Supporting Information:**

**Molecular mechanism of binding between 17β-estradiol and DNA**

Tamsyn A. Hilder^1,2,^* and Justin M. Hodgkiss^1,3,^*

^1^ School of Chemical and Physical Sciences, Victoria University of Wellington, Wellington 6040, New Zealand

^2^ Computational Biophysics Group, Research School of Biology, Canberra, ACT 0200, Australia

^3^ The MacDiarmid Institute of Advanced Materials and Nanotechnology, New Zealand


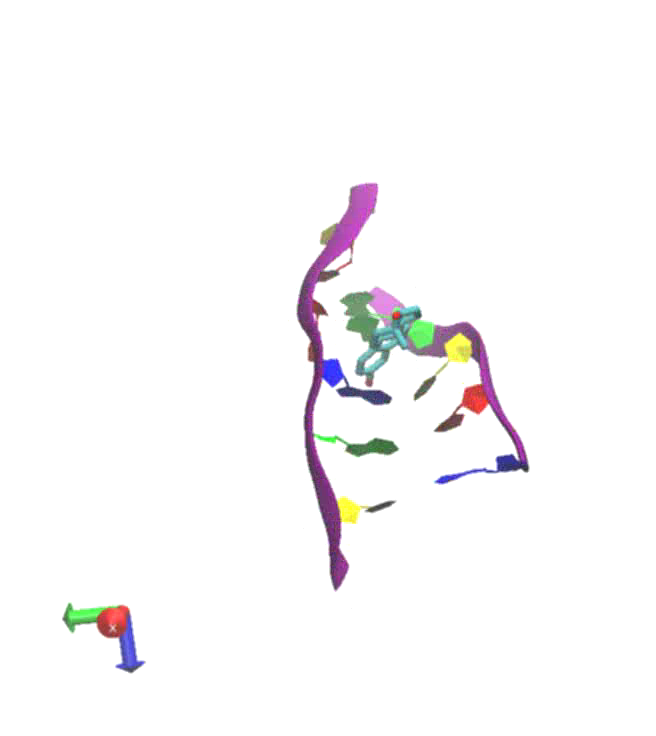


Movie S1. 17β-estradiol moving from its initial ZDOCK bound state to its intercalated state from molecular dynamics simulations. 17β-estradiol is shown in licorice with hydrogen atoms removed for clarity. Only the segment of the DNA strand involved in binding is shown.


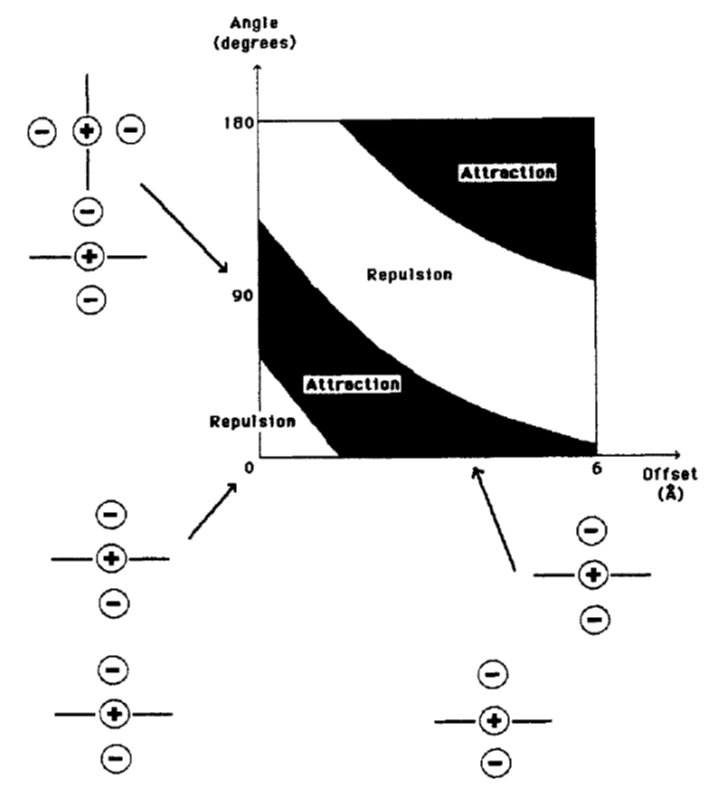


Figure S1. Interaction between two idealized pi-atoms as a function of orientation. Reprinted with permission from [J. Amer. Chem. Soc. 1990, 112: 5525-5534]. Copyright 1990 American Chemical Society.


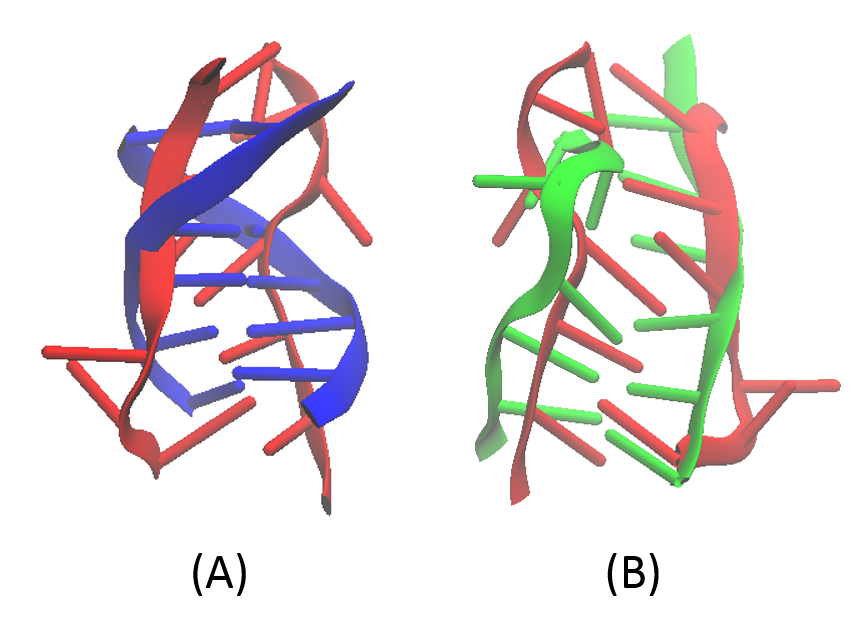


Figure S2. Comparison of DNA structures during simulations. (A) The crystal structure (1HCQ) prior to (blue) and after 40 ns (red) molecular dynamics simulations in the absence of E2. (B) The erDNA structure in the absence (red) and presence of 17β-estradiol after 40 ns of molecular dynamics simulations. For clarity only the portion of the DNA sequence which is involved in binding is shown, at the location of the consensus half site – bases 3 to 8 from Table 1.


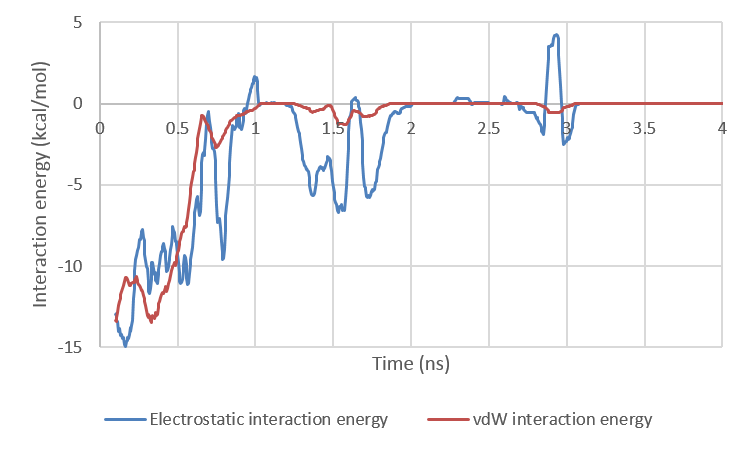


Figure S3. Electrostatic and Van der Waals (VdW) interaction energies between aspirin, and the ds-erDNA. Moving average is displayed, averaging over 20 data points.
